# Developmental Neurotoxicity: Some Old and New Issues

**DOI:** 10.5402/2012/814795

**Published:** 2012-06-24

**Authors:** Gennaro Giordano, Lucio G. Costa

**Affiliations:** ^1^Department of Environmental and Occupational Health Sciences, University of Washington, 4225 Roosevelt Way NE, Suite 100, Seattle, WA 98105, USA; ^2^Department of Human Anatomy, Pharmacology and Forensic Science, University of Parma Medical School, 43121 Parma, Italy

## Abstract

The developing central nervous system is often more vulnerable to injury than the adult one. Of the almost 200 chemicals known to be neurotoxic, many are developmental neurotoxicants. Exposure to these compounds *in utero* or during childhood can contribute to a variety of neurodevelopmental and neurological disorders. Two established developmental neurotoxicants, methylmercury and lead, and two classes of chemicals, the polybrominated diphenyl ether flame retardants and the organophosphorus insecticides, which are emerging as potential developmental neurotoxicants, are discussed in this paper. Developmental neurotoxicants may also cause silent damage, which would manifest itself only as the individual ages, and may contribute to neurodegenerative diseases such as Parkinson's or Alzheimer's diseases. Guidelines for developmental neurotoxicity testing have been implemented, but there is still room for their improvement and for searching and validating alternative testing approaches.

## 1. Introduction

Neurotoxicity has been defined as “any adverse effect on the chemistry, structure or function of the nervous system, during development or at maturity, induced by chemical or physical influences” [1]. An adverse effect is “any treatment related change which interferes with normal function and compromises adaptation to the environment” [2]. Thus, most morphological changes such as neuronopathy (a loss of neurons), axonopathy (a degeneration of the neuronal axon), myelinopathy (a loss of the glial cells surrounding the axon), or other gliopathies, would be considered adverse, even if structural and/or functional changes were mild or transitory. In addition, neurochemical changes, also in the absence of structural damage, should also be considered adverse, even if they are transient and reversible, as they lead to impaired function. The general definition of neurotoxicity also points out to a potential difference between the developing and the mature nervous system, to underscore the fact that developmental neurotoxicity is an important aspect of neurotoxicology. Many known human neurotoxicants are indeed developmental neurotoxicants [3], and in most, but not all cases, the developing nervous system is more sensitive to toxicants than the adult nervous system. Neurotoxicity can also occur as a result of indirect effects, such as damage to hepatic or cardiovascular structures, or because of interference with the endocrine systems. Some chemicals may have multiple modes of action and may affect the nervous system both directly and indirectly. For example, some halogenated compounds may interact directly with brain cells, and also affect the development of the nervous system by altering thyroid hormone homeostasis [4, 5].

## 2. Testing for Neurotoxicity and Developmental Neurotoxicity

Though neurotoxic effects may be detected in the course of standard toxicity testing (e.g., acute, chronic, or developmental/reproductive toxicity studies), specific guidelines exist to further probe the potential neurotoxicity of chemicals [6, 7]. The USEPA (United States Environmental Protection Agency) guidelines focus on a functional observational battery, on measurements of motor activity, and on neuropathological examinations [7]. The OECD (Organization for Economic Co-operation and Development) guidelines similarly focus on clinical observations, functional tests (e.g., motor activity, sensory reactivity to stimuli), and on neuropathology [6]. These batteries are meant to provide a Tier 1 screening for potential neurotoxicity, with positive findings to be followed up by further testing (Tier 2) which may include specialized behavioral tests, electrophysiological and neurochemical measurements, and additional morphologic studies. Examples are tests for measuring learning and memory, measurements of nerve conduction velocity, and biochemical parameters related to neurotransmission or to indices of cell integrity and functions [1]. 

Separate guidelines for developmental neurotoxicity (DNT) testing have also been developed both in the USA and in Europe [8, 9]. Exposure to the test chemicals is from gestational day 6 to postnatal day 10 or 21 to the mother, thus ensuring exposure *in utero* and through maternal milk. Tests involve measurements of developmental landmarks and reflexes, motor activity, auditory startle test, learning and memory tests, and neuropathology. As for neurotoxicity testing, DNT testing has been proven to be useful and effective in identifying compounds with developmental neurotoxicity potential [10]. This is not to say that current DNT testing guidelines cannot be improved; indeed it has been pointed out that they may be overly sensitive and produce a high rate of false positives [11], or, in contrast, that they may be too insensitive and not enough comprehensive [12]. Furthermore, issues have been raised regarding historical control data, toxicokinetic parameters, maternally mediated toxicity versus direct effects, selection of tests and their analysis and interpretation, and others [13, 14].

In the past several years, the need to develop acceptable alternatives to conventional animal testing has been increasingly recognized by toxicologists, to address problems related to the escalating costs and time required for toxicity assessments, the increasing number of chemicals being developed and commercialized, the need to respond to recent legislations (e.g., REACH (Registration Evaluation and Authorization of Chemicals) and the Cosmetics Directive (76/768/EEC) in the EU), and efforts aimed at reducing the number of animals used for toxicity testing. Hence, also in the field of DNT, efforts have been directed toward the development of alternative models, utilizing either mammalian cells *in vitro* or nonmammalian model systems (e.g., zebrafish or **C. * elegans*), which could serve as tools for neurotoxicity and developmental neurotoxicity testing, particularly for screening purposes [15]. These alternative tests should serve as Tier 1 tests to allow the screening of compounds whose potential DNT is unknown. Given the complexity of the nervous system and the multiple facets of possible neurotoxic effects, it is highly unlikely that a single test (as the Ames test for mutagenicity) will cover the spectrum of neurotoxicity. Rather, a battery of tests should be considered which may include some *in vitro* tests with mammalian cells and one or two tests with nonmammalian models. This may be complemented by quantitative structure-activity relationship- (QSAR-) based computational approaches. Novel approaches, part of the “omics” technologies, may also find a role in such endeavor. Alternative models for DNT should attempt to mimic several processes that may occur *in vivo*, and given the complexity of the central nervous system (CNS), the scenario for DNT is much more complex than that for other target organs of toxicity [15].

## 3. Vulnerability of the Developing Nervous System

Several lines of evidence suggest that the developing nervous system may be more susceptible, and/or differentially susceptible, to toxic insult than the adult nervous system. Different parts of the CNS develop at different stages; cell proliferation, migration, and differentiation contribute to the formation of definite brain structures, in which the correct number of cells in the proper location is necessary for proper function [16, 17]. Even within a single brain region, subpopulations of neurons may have different rate of development; for example, in the cerebellum, Purkinje cells develop early (embryonic days 13–15 in the rat, corresponding to gestational weeks 5–7 in humans), while granule cells are generated much later (postnatal days 4–19 in the rat, corresponding to gestational weeks 24–36) [16]. Failure in cell proliferation or cell migration because of exposure to toxic insults (e.g., irradiation or methylmercury) has profound deleterious effects on the developing brain [18]. Though neurons maintain the ability to make new synapses throughout life, the period of brain development when synaptogenesis occurs is critical for the formation of the basic circuitry of the nervous system [19]. Furthermore, in the developing nervous system, neurotransmitters may have function other than neurotransmission, such as modulation of cell proliferation, survival, and differentiation [20]. Thus, any toxicant that interferes with neurotransmission during development may cause permanent defects in the CNS. 

Neurogenesis produces more neurons than those found in the mature nervous system, and excess neurons are pruned by finely regulated apoptotic processes at different developmental times [21]. Any chemical interfering with apoptotic processes may trigger degeneration of neurons that would not otherwise have been deleted or may promote survival of unnecessary cells [22]. In addition, pruning, defined as loss of synapses, also occurs physiologically in the developing brain, and chemicals interfering with this process (which is longer lasting than neuronal loss to apoptosis) would have most significant adverse effects on brain functions [23]. While all these considerations relate to the development of neurons, it has become apparent that glial cells (astrocytes, oligodendrocytes, and microglia) also play a relevant role in brain development and may be the target of toxic action [24]. Indeed, several chemicals (e.g., alcohol, nicotine, and certain pesticides) exert profound neurotoxic effects when exposure occurs during the brain growth spurt, characterized by extensive glial cell proliferation and maturation. In addition to all sensitive processes described, the developing brain is distinguished by the absence of a blood-brain barrier. The development of this barrier is a gradual process, beginning *in utero* and reaching completion around postnatal month six in humans [19]. The incomplete development of the blood-brain barrier allows several endogenous and exogenous chemicals, normally excluded from the brain, to freely enter the developing brain.

## 4. Developmental Neurotoxicants

There are approximately 200 chemicals which have been found to be neurotoxic in humans [3], and for many more there is at least some evidence of neurotoxicity deriving from animal studies. Of these, several are developmental neurotoxicants. However, of over 80,000 chemicals on the market, only a handful (about 200) have undergone developmental neurotoxicity testing according the established guidelines [10] As said, the developing brain is often more sensitive than the adult brain to toxic insult; thus, neurotoxicity is observed at much lower exposure levels. This is the case, for example, of lead or methylmercury. In several cases, the effects of developmental chemical exposure are different from those observed in adult, upon similar exposure; this is the case, for example, of ethanol or valproate. In some cases, developmental exposure to neurotoxicants results in morphological alteration of the CNS, with accompanying changes in functions [18]. However, in several instances, functional changes may be the result of more subtle biochemical/molecular alterations without major structural abnormalities.  Table 1 presents a list of some major developmental neurotoxicants, with indications on findings in animals and humans. Exposure to chemicals which may adversely affect the nervous system has been suggested to be associated with a number of developmental disabilities (learning disabilities, attention-deficit hyperactivity disorder, dyslexia, sensory deficits, mental retardation, and autism spectrum disorders) which are diagnosed in children at an alarming increasing rate [3, 25, 26].

A few selected examples of developmental neurotoxicants are discussed in this review. These include two old, well established compounds (methylmercury and lead), and two classes of chemicals (polybrominated diphenyl ethers and organophosphates) which have been suggested in recent years to cause developmental neurotoxicity. Additional information may be found in other reviews published in recent years [3, 18, 25, 26]. We apologize with several colleagues whose work cannot be cited because of space limitations.

## 5. Methylmercury (MeHg)

This organometal is probably one of the most studied developmental neurotoxicant, because of several episodes of human poisoning over the years, and the fact that low-level exposure, mainly through the consumption of contaminated fish, continues to these days [27–29]. With the possible exception of a case in Sweden in the early 1950s, due to consumption of MeHg-contaminated seed grain, the first evidence of the deleterious effects of MeHg exposure on brain development emerged from Japan in the mid 1950s [30]. Children born from mothers living around Minamata Bay which consumed MeHg-contaminated fish, presented severe neurological deficits, while their mothers appeared unaffected or suffered only mild symptoms [31]. About a decade later, another extensive episode of poisoning occurred in Iraq, due to consumption of MeHg-contaminated grain [32]. In both cases, notable differences were found in the distribution of pathological changes in the young, exposed *in utero* or as children, and in adults [33]. In particular, while damage in adults is restricted to the cerebellum and the visual cortex, diffuse damage is seen in the developing brain [34]. It was estimated that the nervous system during early development *in utero* has a five-fold greater vulnerability to MeHg [35]. Signs and symptoms in MeHg-poisoned children included spastic paresis, mental retardation, movement disorders, seizures, primitive reflexes, and speech difficulty [28, 31]. The mechanisms of MeHg developmental neurotoxicity have been studied extensively, and a very complex picture has emerged. MeHg binds with high affinity to sulfhydryl groups, which are relevant for the proper functioning of a large number of proteins. As such, several key cellular processes are affected by MeHg [18, 27, 29]. For example, MeHg has been reported to cause apoptotic cell death, to cause retraction of growth cones and extension, to impair the cytoskeleton, to affect cell's energetics metabolism, and to reduce cell proliferation and neuronal migration. 

The ban of alkylmercury compounds for use as fungicides and stricter controls on fish contamination has avoided further catastrophic events as those of Japan and Iraq. However, low-level contamination of fish is persistent and may be responsible for developmental neurotoxic effects, particularly in populations with high seafood consumption. Three major longitudinal studies have examined the potential effects of low-level MeHg exposure in New Zealand, the Seychelles, and the Faroe Islands [35–37]. In all locations, MeHg exposure is entirely due to diet, which consists mainly of marine animals. Two of these studies (in New Zealand and the Faroe Islands) reported a correlation between maternal levels of MeHg and subtle neurobehavioral deficits in the offspring. In particular, a small decrease in IQ points and deficits in memory attention and visuospatial perception were noted in both studies [35, 36]. In the Seychelles study, such relation between MeHg exposure and neurodevelopmental effects was not found [38]. Concomitant exposure to polychlorinated biphenyls (PCBs) in the Faroe Islands population, because of consumption of whale meat and whale bubbler, may be an important confounder, as both may have independent neurological effects in this population [39, 40]. Another important confounder in these and in more recent studies [30] is that fish consumption has also beneficial effects for the developing brain, which are ascribed to omega-3 fatty acids [41, 42]. Thus, a balance exists between adverse (MeHg) and beneficial (omega-3) effects, which may dampen toxicity or obscure benefits. As MeHg contamination of fish is difficult to avoid, the best choice for the pregnant consumer is to choose fish species with minimal MeHg burden and relatively high omega-3 content (e.g., sardine, salmon), in order to minimize risk and maximize benefits [42]. Exposure limits for MeHg have been set, with provisional tolerable weekly intake values ranging from 0.7 to 1.6 ug/kg, depending on the regulatory agency [42].

## 6. Lead (Pb)

Pb was discovered thousands of years ago and has been used extensively ever since. In the 1800s, commercial use of Pb increased further, as it was found to be highly effective in paints. In the early 1920s, an organic form of Pb (tetraethyl lead) was found to be very effective as an antiknocking agent in gasoline, starting the long successful sale of leaded fuels. The toxic effects of Pb, however, did not go unrecognized. In recent decades, several efforts to reduce the use of Pb (e.g., banning of Pb paints in the 1970s, phasing out of leaded gasoline in the 1980s) significantly curtailed Pb contamination; yet Pb remains a major, ubiquitous, ecosystem pollutant. Pb is a neurotoxic metal both in adults and in children. In adults, the main effects of Pb poisoning are a peripheral neuropathy, precisely a myelinopathy, which is reversible upon chelation and cessation of exposure. At higher concentrations (100 ug/dL in blood), an encephalopathy can also develop. In contrast, the developing CNS is exquisitely sensitive to the effects of Pb, even at much lower blood levels. In the 1970s, the blood Pb action level in children was 60 ug/mL, the level associated with clinical signs of toxicity in adults [26]. In those years, epidemiological studies clearly showed an association between body burden of Pb in children and adverse neurobehavioral outcomes, namely, lower academic performance and shortened attention span [43]. The phasing out of leaded gasoline and the limitation on smokestack Pb emissions caused Pb blood level in children in the USA to decrease by 80% in the period 1978–1991. Over the years, the level of concern for blood Pb have decreased to 25 ug/mL, then in 1991 to 10 ug/mL, where it stands today. However, blood Pb levels as low as 2 ug/mL have been associated with declines in IQ and various adverse behavioral effects [44, 45], and there is widespread belief that there is no proven safe lower limit for Pb exposure [46, 47]. 

Animal studies in multiple species have confirmed that developmental Pb exposure causes similar cognitive dysfunctions, learning impairment, and distractibility [48]. Of interest is that studies in rodents have shown that early environmental enrichment antagonizes some of the learning and memory deficits resulting from low level exposure to Pb during gestation [49]. Pb has been shown to exert neurotoxicity during differentiation and synaptogenesis [50]; however, the greatest adverse effects are seen during the latest stages of brain development, suggesting that Pb may interfere with the apoptotic process and the trimming/pruning of synaptic connections [51]. *In vivo* and *in vitro* studies have shown that Pb may disrupt the blood-brain barrier by injuring astrocytes, with a secondary damage to the endothelial microvasculature [52]. Developmental Pb exposure has been shown to target the hippocampus, cerebral cortex, and cerebellum [53]. At the molecular level, Pb is known to interfere with the regulatory action of calcium in cell functions. Pb is able to increase intracellular calcium concentrations and serves as a calcium substitute, and some calcium-binding proteins are capable of binding Pb [54]. One important enzyme shown to be activated by low concentrations of Pb is protein kinase C (the classical isoforms), with ensuing perturbations of cellular homeostatic mechanisms including cell proliferation [55, 56]. 

## 7. Polybrominated Diphenyl Ethers (PBDEs)

PBDEs are additive flame retardants, widely used in recent years in a variety of consumer products. Since PBDEs are not chemically bonded to the polymer product, they leach out into the environment and have become persistent organic pollutants, having been detected in most biota, as well as in human blood and breast milk [57, 58]. PBDEs, which comprise 209 possible congeners, have been marketed as mixtures of penta-, octa-, or deca-brominated BDEs. The first two have been banned in the European Union and in several states in the United States, while production of decaBDE will be discontinued soon in the USA [59]. Levels of PBDEs have significantly increased in the past decades, and those in human tissues in North America have been consistently found to be one to two orders of magnitude higher than those reported in Europe and Asia [59, 60]. Particularly alarming is the fact that body burden is the highest in infants (because of exposure through breast milk) and in toddlers (because of exposure through house dust and the diet) [59, 61, 62]. This has raised concerns for the potential developmental toxicity and neurotoxicity of PBDEs [4, 63–67]. Animal studies have provided indications that exposure to different PBDEs (BDE-47 and BDE-99, in particular) during the prenatal and/or postnatal periods causes long-lasting behavioral abnormalities, particularly in the domains of motor activity and cognition (see references in [4, 67, 68]). Toxicokinetic studies also indicate that young mice have a reduced ability to excrete BDE-47, resulting in higher body burden than in adults [69]. 

In humans, in addition to the large database on body burden of PBDEs, evidence is also emerging on possible adverse developmental adverse effects. In a study in Taiwan [70], elevated PBDE levels in breast milk were correlated with lower birth weight and length, lower head and chest circumference, and decreased Quetelet's (body mass) index. In another study in the Netherlands, an association was found between blood PBDE levels in the mother at the 35th week of pregnancy and altered motor function, cognition, and behavior of the child up to age six [71]. In an additional cohort in New York City, prenatal PBDE exposure (as indicated by cord blood PBDE levels) was associated with lower scores on tests of mental and physical development at the ages of 1–4 and 6 years [72]. Two recent additional studies in Spain [73] and in Taiwan [74] reported of neurodevelopmental deficits (decreases in cognitive and motor scores, decreased attention) in infants and children exposed to PBDEs.

The USEPA has proposed Reference Dose (RfD) values for single congeners, based on developmental neurotoxicity (e.g., 200 and 100 ng/kg/day for BDE-47 and BDE-99, resp. [59]). The RfD for BDE-209 (7.0 ug/kg/day), also based on developmental neurotoxicity, has generated some controversy. At difference with other lower brominated BDEs, because of its bulky configuration, BDE-209 is poorly absorbed and does not easily penetrate the cell wall. Though a few animal studies have indicated that BDE-209 may cause developmental neurotoxicity, affecting motor and cognitive domains as other PBDEs, a recent developmental neurotoxicity study, carried out according to international guidelines, has provided no evidence of adverse effects on neurodevelopment [68, 75]. Nevertheless, while estimated exposure to BDE-209 in children appears to be several orders of magnitude below the RfD proposed by the USEPA, questions remain on the extent and relevance of BDE-209 metabolism to lower brominated PBDEs in the environment and in humans [68]. Furthermore, a recent study in Taiwan has found an association between BDE-209 levels in breast milk and cognitive development in infants [74]. 

The mechanisms of PBDEs' developmental neurotoxicity are still elusive, though two general, and not mutually exclusive, modes of action are emerging: one indirect, related to effects of PBDEs on thyroid hormones, and the other involving possible direct effects of PBDEs on the developing brain [4]. Some animal studies have reported alterations of thyroid hormones following developmental PBDE exposures [76], though developmental effects of PBDEs in animals have also been observed in the absence of thyroid hormone alterations [77]. Various *in vitro* studies have investigated the ability of PBDEs to alter cell signaling, particularly protein kinase C and calcium homeostasis, to cause oxidative stress, and to induce apoptotic cell death [78–83].

## 8. Organophosphorus (OP) Insecticides

A report from the National Academy of Sciences first highlighted the potential higher exposure of children to pesticides, and the Food Quality Protection Act indicates that in the risk assessment process, an additional safety factor should be included to ensure protection of children who are presumed to be more sensitive to toxicants' effects [84, 85]. OPs are one of the main classes of insecticides, in use since the mid 1940s. While they are excellent insecticides, OPs, with few exceptions, can also exert significant adverse effects in nontarget species including humans. Their main toxic effect is a cholinergic crisis, due to accumulation of acetylcholine in synapses because of inhibition of acetylcholinesterase (AChE) [86]. Experimental data indicate that the acute toxicity of OPs is influenced by age, with young animals being more sensitive to the effects of exposure [87]. This increased sensitivity is not due to intrinsic differences in AChE, whose catalytic properties are not influenced by age, but rather to lower metabolic abilities of young animals [88]. Depending on the OP, low detoxication by cytochromes P450, by paraoxonase-1, or by carboxylesterase may account for the differential age-related acute toxicity [89, 90]. As these enzymatic systems are believed to show a developmental curve also in humans [91], young children would be expected to be more sensitive than adults to acute OP toxicity. 

An increasing body of literature suggests that developmental exposure to OPs (though most work has been carried out with a single compound, chlorpyrifos), at dose levels causing little inhibition of AChE, results in biochemical and behavioral abnormalities. Experimental studies in rodents indicate that pre- or postnatal exposure to chlorpyrifos affects various cellular processes (e.g., DNA replication, neuronal survival, glial cell proliferation), noncholinergic biochemical pathways (e.g., serotoninergic synaptic functions, the adenylate cyclase system), and causes various behavioral abnormalities (e.g., locomotor skills, cognitive performance) [92–95]. *In vitro* studies have shown that some OPs inhibit astroglial cell proliferation and cause neuronal apoptotic death [96, 97]. These, and other, effects were seen, however, at relatively high OP concentrations, higher then those sufficient to inhibit AChE [98]. In contrast, a few observations report of *in vitro* and *in vivo* effects of OPs at concentrations or doses below those required to inhibit brain AChE catalytic activity [95, 99]. These findings, together with results of biomonitoring studies that indicate exposure of children, particularly in inner cities and in farming communities, to Ops [100, 101], have led to regulatory restrictions on the residential use of certain OPs (e.g., diazinon, chlorpyrifos), and to heightened concern for their potential neurotoxic effects in children [98, 102–104]. For example, a series of studies in different cohorts in New York City and California have reported associations between developmental exposure to chlorpyrifos and other OPs and neurodevelopmental abnormalities in the domains of reflexes and cognitive performance [105–108]. Given their widespread presence, there is certainly the need for a better understanding of the potential developmental neurotoxicity of OPs. If effects are secondary to AChE inhibition, careful consideration of this endpoint must be undertaken to assure that current exposure limits guarantee the safety of children. In addition, studies should keep addressing possible effects of the parent-compound and/or its metabolites, unrelated to AChE inhibition, due to interference with possible noncholinergic targets at low concentrations [88, 98, 108].

## 9. Silent Neurotoxicity, Indirect Neurotoxicity, and Long-Term Effects

Manifestations of neurotoxic effects upon developmental exposure either *in utero*, during lactation, or in early childhood, are usually seen shortly after exposure. Yet, evidence is emerging that deleterious effects of toxicants may not become clinically evident for some months, or even several years after exposure. This period during which the individual may manifest no evidence of toxicity is referred to as the “silent” period [109]. Silent toxicity is defined as persistent biochemical or morphological injury which remains clinically unapparent unless unmasked by experimental or natural processes [109, 110]. Silent toxicity may be compared to the process of carcinogenesis, in which molecular and cellular damage occurs years if not decades before any clinical manifestation of the disease [111]. In case of neurotoxicity, an example of silent toxicity is represented by the Parkinsonism-dementia known as Guam's disease, in which latencies of decades have been reported before alleged still undefined exposures and clinical signs [112]. Similarly, bovine spongiform encephalopathy (mad cow disease), a variant of Creutzfeld-Jacob disease, has been shown to have a latency of decades [113]. The delay between exposure and clinical expression of neurotoxic injury may be ascribed to various causes. For example, a selected population of neurons may be affected, but the known plasticity of the brain would compensate for such loss. However, further exposure to exogenous influences (e.g., stress, disease, additional chemical exposure), or the natural aging process, would unmask the existing deficit. Alternatively, the organism may be initially able to compensate for a certain deficit, but progressive loss of function would eventually overcome the functional reserve and plasticity of the brain [109].

The possibility that such latent period between exposure and clinical manifestation would occur in the context of development is even more probable. The concept that adult disease may have a fetal origin has been introduced by David Barker and is known as the “Barker hypothesis” [114]. Exposure to chemicals may cause direct damage or alter developmental programming, whose resulting functional deficits become apparent later in life. A famous example is that of diethylstilbestrol, in which *in utero* exposure leads to an increase in vaginal adenocarcinoma around the time of puberty [115]. In case of developmental neurotoxicity, an example is represented by the findings of experimental studies on prenatal infection. Perinatal exposure of rats to the Gram (−) bacteriotoxin lipopolysaccharide causes a 30% loss in dopaminergic neurons in the substantia nigra and persistent injury to the dopaminergic system [116, 117], suggesting that, in humans, prenatal infections occurring at the appropriate gestational age would result in the birth of an individual with fewer dopaminergic neurons. This would be initially inconsequential, as clinical signs of Parkinson's disease are not apparent until about 80% of dopaminergic neurons are lost. However, this early-life damage would predispose an individual to develop Parkinson's disease as the aging process brings along a normal progressive loss of dopaminergic neurons [118]. Developmental exposures to certain pesticides, such as the herbicide paraquat and the fungicide maneb, which also target dopaminergic neurons, have also been implicated in the later development of Parkinson's disease [119]. Similarly, developmental exposure to the now banned organochlorine insecticide dieldrin has been shown to cause long-lasting alterations of the dopaminergic system, typical of a silent dopaminergic dysfunction [120].

In some occasions, early mild damage may worsen as the individual matures and ages. For instance, neurotoxic effects of developmental exposure to MeHg may be unmasked only during aging [121, 122]. *In utero* exposure to methylazoxymethanol, which causes microencephaly, caused a premature decline in cognitive functions [123], and the neurotoxic effects of neonatal exposure of triethyltin, a glial neurotoxicant, were exacerbated by aging [124]. In other situations, this may not be the case, yet the neurotoxic effects of developmental exposure appear to be irreversible, and even if they do not worsen with age, they are certainly long-lasting, as shown, for example, for PCB-126 [125]. Perturbations of cognitive functions due to developmental Pb exposure remain for life [126], and perinatal Pb exposure has been associated with the development of schizophrenia and of Alzheimer's disease [127–129]. It has been suggested that exposure to Pb during critical periods of brain development may impact the expression and regulation of amyloid precursor protein later in life, potentially altering the course of amyloidogenesis, thereby acting as a risk factor for the onset of Alzheimer's disease-like pathology [128, 130, 131].

The development of the nervous system can also be influenced by chemicals which may not interact directly with neuronal and glial cells but may act instead, or in addition, as endocrine disruptors. For example, as indicated in a previous section, PBDEs are thought to exert developmental neurotoxicity by two general, and not mutually exclusive, modes of action, one related to effects on thyroid hormones, and the other involving direct effects of PBDEs on the developing brain [4]. Thyroid hormones are known to play a relevant role in brain development [132], and hypothyroidism has been associated with a large number of neuroanatomical and behavioral effects [133, 134]. PBDEs have been shown to perturb the thyroid system both during development, leading to a reduction of circulating thyroid hormone [76]. Though the exact mechanism(s) underlying these effects are unclear, reduction in thyroid hormones may contribute to developmental neurotoxicity of PBDEs, though effects have been seen also in the absence of thyroid hormone changes [77]. Experimental compounds such as propyl thiouracyl (PTU), which inhibits thyroid hormone synthesis and causes hypothyroidisms, are clear developmental neurotoxicants, as evidenced by behavioral and morphological alterations in the CNS [134, 135]. Decreases of T_4_ following developmental exposure to PBDEs or PCBs range from 10 to 60%. As decrements in neurological development in children of mothers with 25% decrease in T_4_ have been reported [133], the effects of these chemicals on thyroid hormones may well contribute to their developmental neurotoxicity.

In addition to chemicals affecting thyroid hormones, other endocrine disruptors may also exert adverse effects on the developing nervous system [136]. Much attention has for example been devoted to bisphenol-A, a plasticizer used in several industrial and consumer products, as several studies have shown that this compound when given perinatally to experimental animals, causes various behavioral and morphological alterations [137–139]. In contrast to these findings, however, a developmental neurotoxicity test, done according USEPA and OECD guidelines, provided negative results [140]. 

## 10. Conclusion, New Directions, and Research Needs

Both prenatal and early postnatal exposure to neurotoxic chemicals may have deleterious influences on the structure and functions of the nervous system. It has been estimated that one out of six children has a developmental disability, and in most cases these disabilities affect the nervous system [3]. These include learning disabilities, autism-related disorders, attention deficit hyperactivity disorder, developmental delays, cerebral palsy, or sensory deficits. Such disorders are usually difficult to correct either pharmacologically or by other types of intervention and are thus permanent, causing enormous damage and costs to families and society. While there are many etiological causes to such developmental disabilities, it has been estimated that around 3% may be due exclusively to exposure to neurotoxicants, and another 25% may arise from the interaction of individual genetic susceptibility and environmental chemicals [141]. It has been often underlined that even more subtle changes, such as a decrease of a few IQ points, may not have a significant impact on a single individual, but are of great significance for society, as would be the case of Pb [46]. Whether developmental exposure to neurotoxicants may accelerate CNS disturbances associated with aging is still being investigated; at the same time initial experimental evidence points out to the issue of silent neurotoxicity, subclinical alterations which, when superimposed to the normal aging process or to additional insults, would result in frank neurotoxicity, such as neurodegenerative diseases.

While there is increasing recognition of the relevance of DNT, there are still several areas of research that would deserve further investigations. First, there is the issue of identifying mechanism(s) of DNT. This is no easy task, as multiple mechanisms are undoubtedly operational for most neurotoxicants. The unraveling of mechanisms should not be seen as a pure academic exercise; while it is true that identification of specific mechanisms is not necessary for risk assessment (the cases of MeHg and Pb are classical examples), knowledge of mechanisms may serve at developing targeted therapeutic intervention strategies. Additionally, testing guidelines for developmental neurotoxicity may have to be revisited, as situations like those mentioned in this article (e.g., for BDE-209 and bisphenol-A for which guideline developmental neurotoxicity testing provided no evidence of adverse effects, in contrast to other findings), complicate a proper health risk evaluation. In this regard, much effort should also address the development and validation of novel batteries of alternative DNT tests. Finally, the need remains to establish potential causal associations between chemical exposures and developmental abnormalities in humans, through rigorous epidemiological studies.

## Figures and Tables

**Table 1:** Examples of developmental neurotoxicants.

Category	Compound	Evidence of DNT	
		Animals	Humans
	Methylmercury	+++++	+++++
Metals	Lead	+++++	+++++
	Manganese	+++	+++
	Arsenic	+++	++

Solvents	Ethanol	+++++	+++++
	Toluene	++++	+++++

	Organophosphates (various)	++	++
Pesticides	Organochlorines (dieldrin)	+	−−
	Herbicides (paraquat)	+	−−
	Fungicides (maneb)	+	−−

	PCBs	++++	+++
	PBDEs	+++	+
Other contaminants	Phtalates	++	++
	Bisphenol A	++	−−
	PFOS/PFOA	+	−−

Natural toxins	Domoic acid	++	−−

Pharmaceutical drugs	Antiepileptic compounds	++++	++++

Drugs of abuse	Cocaine	++	++

PCBs: polychlorinated biphenyls, PBDEs: polybrominated diphenyl ethers, PFOS/PFOA: perfluorooctane sulfate/perfluorooctanic acid. Symbols (+) are arbitrary evaluations by the authors.
